# Evaluation of Remnant Esophageal Perfusion Using Indocyanine Green Fluorescence Imaging during Ivor Lewis Esophagectomy after Total Pharyngolaryngectomy: A Case Report

**DOI:** 10.70352/scrj.cr.25-0737

**Published:** 2026-02-21

**Authors:** Shirou Kuwabara, Kazuaki Kobayashi, Natsuru Sudo, Masanori Nobuhiro, Gen Tomizawa, Yoh Kajiyama, Kohei Sato, Jun Yamamoto

**Affiliations:** Department of Digestive Surgery, Niigata City General Hospital, Niigata, Niigata, Japan

**Keywords:** esophageal cancer, total pharyngolaryngectomy, indocyanine green fluorescence imaging, Ivor Lewis esophagectomy

## Abstract

**INTRODUCTION:**

Head and neck cancer frequently coexists with synchronous or metachronous esophageal cancer, owing to shared carcinogenic exposures such as alcohol and tobacco use. In patients undergoing esophagectomy after total pharyngolaryngectomy (TPL), surgical reconstruction poses significant challenges due to dense adhesions and altered cervical anatomy. The McKeown procedure requires cervical manipulation, demanding careful preservation of the tracheal stoma and free jejunal graft blood supply. In contrast, the Ivor Lewis procedure eliminates the need for cervical maneuver but necessitates an intrathoracic anastomosis between the gastric conduit and the remnant esophagus. This raises concerns regarding potential ischemia of the remnant esophagus, particularly after prior TPL, in which proximal blood supply may be compromised. We report a case of successful robot-assisted Ivor Lewis esophagectomy after TPL, in which intraoperative indocyanine green (ICG) fluorescence imaging was used to confirm sufficient blood supply in the remnant esophagus and to ensure a safe anastomosis.

**CASE PRESENTATION:**

A 73-year-old man with a history of TPL for hypopharyngeal cancer, endoscopic submucosal dissection for gastric and esophageal cancer, and colon resection for descending colon cancer was diagnosed with a new esophageal squamous cell carcinoma during routine surveillance endoscopy. Contrast-enhanced CT revealed no lymph node or distant metastasis (cT1bN0M0, Stage I). Considering his prior TPL and the absence of cervical lymph node involvement, a robot-assisted Ivor Lewis esophagectomy with intrathoracic gastric conduit reconstruction was planned without cervical manipulation. Intraoperative ICG fluorescence imaging demonstrated adequate perfusion of the remnant esophagus (about 45 mm), confirming its viability and allowing safe esophagogastric anastomosis. The postoperative course was uneventful, and the patient was discharged on POD 12. Histopathological examination revealed moderately differentiated squamous cell carcinoma invading the lamina propria mucosa (pT1aN0M0, Stage IA). The patient remains disease-free 2 years postoperatively.

**CONCLUSIONS:**

Ivor Lewis esophagectomy can be safely performed after TPL when careful intraoperative evaluation of remnant esophageal perfusion is undertaken. ICG fluorescence imaging provides a simple and reliable method for assessing blood supply, helping to prevent ischemic complications. This technique may expand the surgical options for patients requiring esophagectomy following TPL.

## Abbreviations

ESD
: endoscopic submucosal dissection
ICG
: indocyanine green
ITA
: inferior thyroid artery
TPL
: total pharyngolaryngectomy

## INTRODUCTION

Head and neck cancer and esophageal cancer share common carcinogenic risk factors, most notably tobacco use and alcohol consumption. This relationship has been conceptually described by Slaughter et al. through the theory of field cancerization,^1)^ which states that chronic exposure of the upper aerodigestive tract to these carcinogens results in widespread epithelial injury and the development of multiple primary malignancies. Consequently, patients with head and neck cancer have a high risk of developing synchronous or metachronous esophageal cancer. Conversely, patients with esophageal cancer are also at increased risk of developing synchronous or metachronous head and neck cancer.

In recent decades, advancements in the diagnosis and treatment of head and neck cancer have led to improved long-term survival. As a result, the incidence of metachronous second primary tumors, particularly esophageal cancer, has become increasingly evident. Previous studies have reported that the cumulative incidence of esophageal cancer after curative treatment for head and neck cancer ranges from 8.5% to 12.1% at 5 years and from 11.2% to 16.5% at 10 years.^2)^ Among the various subtypes of head and neck cancer, patients with squamous cell carcinoma, especially hypopharyngeal carcinoma, are at a particularly high risk for subsequently developing esophageal cancer. Esophageal surgery in patients with a history of head and neck cancer, particularly after TPL, requires careful consideration of cervical and cervicothoracic adhesions, anastomotic site selection, the use of free jejunal grafts, and the blood supply to the remnant esophagus. In recent years, ICG fluorescence imaging has gained attention for its utility in the intraoperative assessment of tissue perfusion, and its value has also been demonstrated in esophageal surgery. Herein, we report a case of esophageal cancer after TPL that was successfully reconstructed using the Ivor Lewis procedure, in which ICG fluorescence imaging was employed to evaluate the blood perfusion of the remnant esophagus, thereby enabling safe reconstruction.

## CASE PRESENTATION

**Fig. 1:**
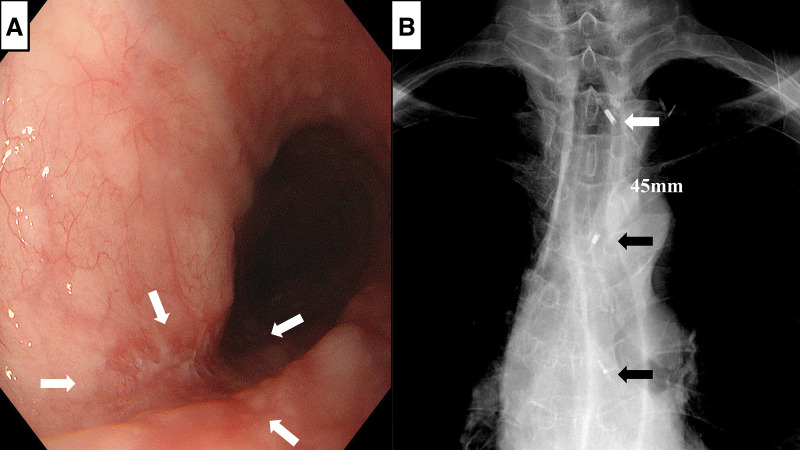
Upper gastrointestinal endoscopy and chest X-ray following endoscopic clipping. (**A**) Upper gastrointestinal endoscopy showing squamous cell carcinoma in the middle thoracic esophagus (arrowheads). (**B**) Marking clips were placed at the site of the jejunal–esophageal anastomosis (white arrowhead) and on both the proximal and distal sides of the esophageal carcinoma (black arrowheads). The distance between the jejunal–esophageal anastomosis and the proximal side of the esophageal carcinoma was approximately 45 mm.

A 73-year-old man underwent screening esophagogastroduodenoscopy, which revealed a depressed lesion located 25–29 cm from the incisors. Biopsy confirmed moderately differentiated squamous cell carcinoma (**Fig. 1A**). Contrast-enhanced CT showed no evidence of lymph-node or distant metastasis, and the clinical stage was determined as cT1bN0M0, Stage I according to the UICC 8th edition.^3)^

The patient’s medical history included descending colon cancer treated with colon resection 24 months earlier, gastric and esophageal cancers treated with ESD 120 months earlier, and hypopharyngeal cancer (pT3N0M0, Stage III) treated with TPL 156 months earlier. The lesion was identified as a depressed-type tumor measuring approximately 4 cm, raising suspicion of submucosal invasion; therefore, ESD was not considered an appropriate treatment option. In addition, the patient had previously experienced severe adverse events related to chemotherapy administered at the time of TPL and consequently declined further chemoradiotherapeutic treatment. As the patient had no significant comorbidities, he was considered an appropriate candidate for radical surgery, and robotic esophagectomy was performed after obtaining informed consent.

### Surgical procedure

Before surgery, marking clips were placed at the jejunal–esophageal anastomosis and on the proximal and distal sides of the esophageal carcinoma. The distance between the jejunal–esophageal anastomosis and the proximal side of the esophageal carcinoma was measured on chest radiography and was estimated at 45 mm (**Fig. 1B**). Considering the patient’s prior TPL, the absence of upper mediastinal and cervical lymph node metastasis, and the preservation of blood flow to the remnant esophagus, cervical manipulation and upper mediastinal lymphadenectomy were omitted. Reconstruction with a gastric conduit in the upper thoracic space using the Ivor Lewis procedure was planned.

### Intraoperative findings

**Fig. 2:**
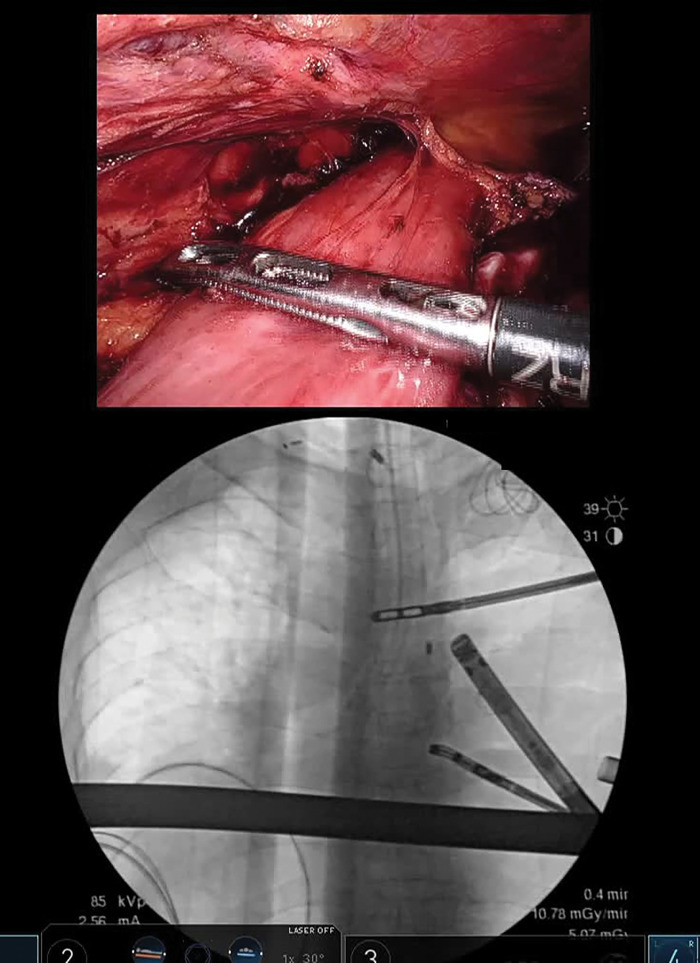
Esophageal transection during surgery. Transection of the middle thoracic esophagus at the proximal margin of the esophageal carcinoma was performed under fluoroscopic guidance using preplaced clips as anatomical landmarks, with forceps indicating the transection line.

Under robotic assistance, a long, greater curvature–based gastric conduit was created in the supine position, followed by middle and lower mediastinal lymphadenectomy in the prone position. Under fluoroscopic guidance, the upper thoracic esophagus was transected using the preoperatively placed clips as landmarks (**Fig. 2**). As measured on preoperative chest radiography, the distance from the transection line to the jejunal–esophageal anastomosis created during TPL was approximately 45 mm, leaving about 45 mm of proximal esophagus. The right bronchial artery and azygos vein arch, located caudal to the transection line, were divided.

**Fig. 3:**
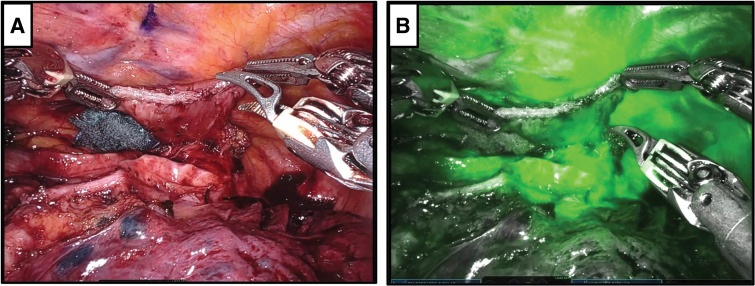
Assessment of blood flow in the remnant esophagus using ICG fluorescence. (**A**) Image showing the remnant esophagus. (**B**) Evaluation of blood flow in the remnant esophagus after intravenous ICG administration, demonstrating good fluorescence in the remnant esophagus. ICG, indocyanine green

Before reconstruction, 3 mg of ICG was administered intravenously, and perfusion of the remnant esophagus was assessed 30 s after injection using the Firefly fluorescence imaging mode (Intuitive Surgical, Sunnyvale, CA, USA). Fluorescence was detected throughout the remnant esophagus within 60 s after injection without focal delay or defects (**Fig. 3** and **Supplementary Video**). Based on this finding, we concluded that the anastomosis could be safely performed and proceeded with an esophagogastric anastomosis using the overlap technique with a linear stapler. The total operative time was 594 min, and intraoperative blood loss was 76 mL.

### Postoperative course

The postoperative course was uneventful, and the patient was discharged on POD 12. Histopathological examination revealed moderately differentiated squamous cell carcinoma measuring 40 × 25 mm, with invasion confined to the lamina propria mucosa and no lymph-node metastasis, resulting in a final pathological diagnosis of pT1aN0M0, Stage IA. At 2 years after surgery, the patient remains free of recurrence.

## DISCUSSION

Head and neck cancer, particularly hypopharyngeal cancer, is frequently associated with synchronous or metachronous esophageal cancer. The standard curative surgery for hypopharyngeal cancer is TPL, while esophagectomy is the mainstay treatment for esophageal cancer. However, when both malignancies occur in the same patient, subsequent surgery becomes technically challenging due to severe adhesions, altered anatomy, and limited reconstruction options, regardless of whether TPL or esophagectomy is performed first. In cases of esophagectomy after TPL, 2 major surgical approaches have been described. The first is the McKeown procedure, in which a gastric conduit is brought to the neck and anastomosed to a free jejunal graft after total esophagectomy. The second is the Ivor Lewis procedure, in which the upper thoracic esophagus is preserved and an intrathoracic esophagogastric anastomosis is performed. Although both techniques have been used in clinical practice, an optimal approach for this complex situation has not been established.

Sugawawa et al.^4)^ and Kashu et al.^5)^ reported favorable outcomes with the McKeown procedure after TPL, with no major complications observed. Conversely, Takahashi et al.^6)^ analyzed 12 cases of Ivor Lewis esophagectomy after TPL and reported no anastomotic leakage. In the McKeown procedure, severe fibrosis of the thoracic inlet and neck following TPL complicates surgical manipulation. Careful attention is required to avoid ischemia or injury to the tracheostoma and to preserve blood supply to the free jejunal graft reconstructed with microvascular anastomosis. These challenges are further intensified in patients who underwent adjuvant radiotherapy. In contrast, the Ivor Lewis procedure avoids a cervical maneuver and is therefore less influenced by fibrosis or radiation-induced tissue change. However, a segment of remnant esophagus, left after both distal and proximal resections, raises concerns about anastomotic leakage due to compromised blood supply. Furthermore, should anastomotic leakage occur, management is generally easier in the McKeown procedure, where the anastomosis is located in the neck, whereas in the Ivor Lewis procedure, which involves an intrathoracic anastomosis, leakage can progress to severe complications such as mediastinitis or empyema.

**Fig. 4:**
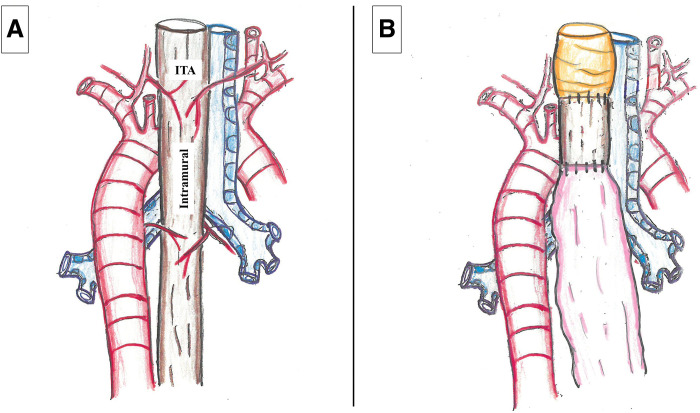
Blood supply to the upper thoracic esophagus (posterior aspect). (**A**) The upper thoracic esophagus is supplied by the ITA, and the intramural supply cranially and caudally along the esophageal wall. (**B**) In the Ivor Lewis procedure following TPL, blood supplies from the ITA and intramural supply in both the cranial and caudal directions are lost. ITA, inferior thyroid artery; TPL, total pharyngolaryngectomy

The upper thoracic esophagus receives blood supply from the ITA and intramural circulation from both cranial and caudal directions.^7)^ In cases of Ivor Lewis esophagectomy after TPL, the ITA and cranial intramural supply are lost, and resection of the esophagus eliminates the caudal intramural blood supply (**Fig. 4**). However, Kuriyama et al.,^8)^ in a comparison of 12 McKeown and 24 Ivor Lewis cases, reported no necrosis or anastomotic leakage in the Ivor Lewis group. Similarly, Okamura et al.,^9)^ in a nationwide survey, found no significant difference in overall survival between the 2 techniques, but noted a significantly lower incidence of anastomotic leakage in the Ivor Lewis group (6.2% vs. 46.7%, P <0.001), supporting the preference for Ivor Lewis reconstruction.

Recent studies have shown that intraoperative ICG assessment of reconstructed organ perfusion significantly reduces anastomotic leakage.^10–12)^ In the present case, the Ivor Lewis procedure was selected due to the absence of lymph node metastasis in the upper mediastinum and cervical region. Intraoperatively, ICG fluorescence imaging confirmed adequate perfusion of the remnant esophagus. To our knowledge, this is the first report to objectively demonstrate remnant esophageal perfusion using ICG fluorescence imaging during an Ivor Lewis procedure after TPL. Although in the reverse surgical sequence, Oshikiri et al.^13)^ similarly demonstrated preserved perfusion in the remnant esophagus during TPL after esophagectomy using ICG.

These findings suggest that the remnant esophagus does not inevitably become ischemic after TPL followed by esophagectomy, or vice versa. Instead, neovascularization from the jejunal–esophageal or esophagogastric anastomosis may develop over time, restoring mural blood supply. Consistent with this concept, Kuriyama et al.^8)^ recommended minimizing dissection around the remnant esophagus to preserve blood supply. The optimal interval between TPL and esophagectomy, as well as the appropriate length of the remnant esophagus, remains unclear. Previous reports by Takahashi et al.^6)^ and Okamura et al.^9)^ described intervals of 32 and 40 months, respectively. However, objective assessment of perfusion in the remnant esophagus is often difficult. In such situations, ICG fluorescence imaging offers a reliable, real-time technique for evaluating esophageal perfusion. Ishii et al.^14)^ also demonstrated that cervical esophageal perfusion assessment with ICG during jejunal reconstruction after esophagectomy reduced anastomotic leakage, emphasizing the importance of confirming perfusion not only in the conduit but also in the remnant esophagus. In our case, omission of upper mediastinal and cervical lymphadenectomy, combined with the long interval of 156 months between procedures, may have contributed to preserving remnant esophageal perfusion.

In our patient, a slightly longer gastric conduit was prepared to enable conversion to the McKeown procedure should remnant esophageal perfusion prove inadequate. If preservation of the previously interposed jejunal graft had not been feasible, reconstruction using a pedunculated gastric tube^15,16)^ was also considered. For esophagectomy following TPL, the Ivor Lewis approach appears to be a reasonable first-line option for middle- to lower-thoracic esophageal cancer when a negative proximal margin can be secured. Intraoperative confirmation of remnant esophageal perfusion using ICG fluorescence allows real-time assessment. However, an important point should be noted in this case: a previous ESD for another esophageal lesion left the remnant esophagus at continued risk for secondary or subsequent carcinogenesis. Therefore, meticulous long-term endoscopic surveillance is required to enable early detection of potential metachronous lesions.

## CONCLUSIONS

Intraoperative ICG fluorescence imaging is a simple, noninvasive, and effective technique for real-time assessment of blood perfusion in the remnant esophagus during Ivor Lewis esophagectomy after TPL.

## SUPPLEMENTARY MATERIALS

Supplementary Video Evaluation of blood flow in the remnant esophagus at 30 s after intravenous administration of ICG, demonstrating good fluorescence in the remnant esophagus within 60 s.

## DECLARATIONS

### Availability of data and materials

All data supporting this article are included in this manuscript.
